# Public Understanding and Attitudes towards Meat Chicken Production and Relations to Consumption

**DOI:** 10.3390/ani7030020

**Published:** 2017-03-09

**Authors:** Ihab Erian, Clive J. C. Phillips

**Affiliations:** Centre for Animal Welfare and Ethics, School of Veterinary Science, University of Queensland, Gatton, Queensland 4343, Australia; ihab.erian@gmail.com

**Keywords:** animal welfare, attitudes, chicken, knowledge, consumption, poultry

## Abstract

**Simple Summary:**

Public knowledge of meat chicken production and how it influences attitudes to birds’ welfare and consumer behaviour is poorly understood. We therefore conducted a survey of the public in SE Queensland, Australia, from which we determined that industry knowledge was limited. Where it existed, it related to an empathetic attitude towards chicken welfare and an increase in chicken consumption. This suggests that consumers who eat more chicken believe that they should understand the systems of production of the animals that they are consuming.

**Abstract:**

Little is known about public knowledge of meat chicken production and how it influences attitudes to birds’ welfare and consumer behaviour. We interviewed 506 members of the public in SE Queensland; Australia; to determine how knowledge of meat chicken production and slaughter links to attitudes and consumption. Knowledge was assessed from 15 questions and low scores were supported by respondents’ self-assessed report of low knowledge levels and agreement that their knowledge was insufficient to form an opinion about which chicken products to purchase. Older respondents and single people without children were most knowledgeable. There was uncertainty about whether chicken welfare was adequate, particularly in those with little knowledge. There was also evidence that a lack of empathy towards chickens related to lack of knowledge, since those that thought it acceptable that some birds are inadequately stunned at slaughter had low knowledge scores. More knowledgeable respondents ate chicken more frequently and were less likely to buy products with accredited labelling. Approximately half of the respondents thought the welfare of the chicken was more important than the cost. It is concluded that the public’s knowledge has an important connection to their attitudes and consumption of chicken.

## 1. Introduction

Consumers’ selection of food is governed by many factors, including culture, religion, lifestyle, diet, knowledge, health concerns and food trends, often influenced by the media [1]. Because they are no longer intimately involved in the food production process, the public’s trust in the product is largely dependent on livestock producers having an empathetic approach to the animals used in the production of food [2]. This involves conforming to ethical standards throughout the breeding, growing and processing of the product. Two important concepts govern the intention to purchase animal-welfare-friendly products: consumer self-identification with ethical issues and Theory of Planned Behaviour, in which the attitudes, subjective norms and perceived level of behavioural control combine to influence the intention to purchase [3]. Self-identification is influenced by socio-demographic factors and the consumer’s animal-related experiences [3]. The latter may be closely linked to their understanding of production systems; we chose meat chicken production to investigate this as it is one of the areas in which there is major concern for animal welfare. It is also important to recognize that consumers may identify animal welfare as far from optimal, but continue to buy and eat meat [4]. This is only possible because of distancing themselves from the production process.

Little is known about the public’s sources of information on animal welfare, including the role of the media, with the associated problem of the accuracy of reporting. Their understanding of animal production systems may be anthropomorphic, an approach supported by some animal welfare scientists [5]. The media may particularly influence public opinion on contentious issues, such as the phasing out of battery cages [6,7]. Conversely, some possible developments in chicken production, such as breeding blind chickens, are not supported by the public because of their interference with bird integrity, even if they do appear to give welfare advantages in intensive production [8]. Many authors have attempted to alert the public to the welfare impact of intensifying production systems, starting with Ruth Harrison in the 1960s [6]. As production systems have intensified, it has become difficult for the public to assess the animals’ welfare [7]. Modern industrial chicken meat production practices are designed to provide low-cost meat to consumers, retailing at less than one-half the price of other meats [9]. Many consumers have a negative perception of intensive farming and say that they are willing to pay more for food produced where animal welfare standards are considered and followed [10,11,12]. However, many consumers do not purchase the products from animals kept in better welfare because of the high price [13].

Despite a belief that welfare is being sacrificed for industrial-scale production, the demand for chicken meat is increasing worldwide; for example, the Australian market has increased by 160% in the last 20 years and the consumption of chicken has exceeded the consumption of any other kind of meat [9]. As well as low cost, the chicken industry attributes the rapid increase of demand to the versatility and ease of handling and cooking chicken products, and the fact that they are a low-fat protein source [9]. However, some discriminatory buying by consumers is evidenced in their reluctance to buy meat produced from intensive systems if the quality of meat produced is perceived to be adversely affected by the way the animals have been treated [14].

The Australian chicken industry has a “vertically integrated” structure. Individual companies control almost all aspects of production: breeding farms, hatcheries, feed mills, supply of feed to contractors, broiler growing farms/units, medication, transport, and initial and further processing plants [9,15]. The management from day-old stock until the day of processing, including staffing, housing and equipment, is mostly contracted out to growers (for example, 800 growers in Australia produce 80% of meat chickens, nearly all under contract to just two integrated national companies [9]. Growers are paid a negotiated monetary return per 100 birds or per weight at the end of the growing cycle. The typical modern unit has 3–10 poultry sheds that are tunnel ventilated, each about 150 × 15 m, with a capacity of 40–60,000 birds [9,16]. Aspects of production that relate to welfare, such as stocking densities, lighting regime and general husbandry practices, are usually determined by the companies’ regulatory quality control systems or the industry code of practice [17]. Apart from contract rearing, other meat chickens are produced by large company farms, or on farms owned and managed by intermediary companies, where each is controlled by a manager who is contracted to a processing company. Breeding farms owned by the major chicken companies are strategically located across Australia, with a trend towards siting of great-grandparent and grandparent breeder farms in areas isolated from traditional poultry rearing places to reduce the risk of exposure to disease agents [9,16].

The aim of this study was to assess the public’s knowledge of chicken production systems and its influence on attitudes towards animal welfare and chicken consumption. We hypothesized that, in line with the Theory of Planned Behaviour, the knowledge of the public about chicken production systems would have an impact on attitudes and the way consumers choose chicken products, and that these would also be influenced by key demographic factors. We anticipated that low levels of knowledge would cause uncertainty in choosing chicken products and in attitudes towards chicken welfare.

## 2. Materials and Methods

We used a quantitative questionnaire that addressed (1) public knowledge of intensive chicken meat production, including transport and slaughtering systems; (2) the attitude of the public towards meat chicken welfare and (3) their choice of chicken products. Socio-demographic questions were also included, which were used to further explain consumer behaviour and identify potential market segments. The questionnaire was designed taking into account the literature on public knowledge and attitudes towards chicken production systems [18,19,20,21,22,23,24]. We defined knowledge as “facts, information, and skills acquired through experience or education; the theoretical or practical understanding of a subject” and attitude as a “relatively stable favourable or unfavourable feeling or belief about a concept, person, or object” [25].

A total of 2663 consumers were approached in a face-to-face survey conducted with respondents who were randomly selected from the public in shopping centres, social clubs, cultural events and professional gatherings in Brisbane CBD, Surfers Paradise on the Gold Coast and a suburb of north Brisbane (Strathpine) during April and May 2013. Locations were selected to obtain a broad spectrum of views, in order to most accurately determine the relationships between knowledge and attitudes/consumption. Only respondents aged 18 years and over were eligible to take part. Clearances were obtained from the Brisbane City Council and the University of Queensland Human Ethics Committee (reference number 2013000458).

### 2.1. Questionnaire Design

A pilot survey was conducted by randomly selecting 15 individuals in Brisbane on each of three successive days. Following this, adjustment was made to the order and language of some of the questions to avoid any possible bias or leading responses.

The final questionnaire focused on the public knowledge and attitude to meat chicken production systems and the consumption of chicken products. Demographic questions were included to determine the respondent’s gender, age, education level, place of residence, income, marital status and religion. Two initial questions addressed subjective knowledge, asking respondents about their level of knowledge about chicken production systems (options: expert, good, some, little or no knowledge) and how they gained it (formal qualifications, farm employment, personal interest, friends and acquaintances and all of these). Then the questions covered three topics specifically related to meat chicken production: (a) objective knowledge of common practices during rearing, transport and slaughter of broilers, how much knowledge they thought they had and whether it was sufficient for choosing which products to purchase, (b) attitudes towards the welfare of birds on farm and during transport and slaughter, and (c) frequency of consumption of different chicken products, their attitude towards labelling systems and willingness to pay more for accredited chicken products (defined as accredited by *FREPA* (Free Range Egg and Poultry Association), *RSPCA* approved farming, *ACO* (Australian Certified Organic) farming, *NASAA* (National Association for Sustainable Agriculture, Australia) or *OGA* (Organic Growers of Australia)). In total there were 15 knowledge questions (Appendix A), 13 attitude questions, four consumption questions and 10 demographic questions. In the knowledge section, there were eight initial questions, all of which were marked correct (score 1) or incorrect (score 0), except one which asked the normal distance that chickens travel from their place of rearing to the abattoir. Respondents were given a score of 1 for a distance of 5–100 km, this being normal in Australia, and 0.5 for a distance of 100–200 km. Respondents were also asked how long it takes intensively-reared meat chickens to reach a slaughter weight of 2 kg (1 point for 35–45 days; 0.5 point for 30–35 days, otherwise no score). We were also able to explore the relationship between knowledge score and respondents’ self-reported understanding of chicken production systems as a validity check. Participants were asked to express their three biggest welfare concerns during transport and in production barns, with each valid welfare concern given a score of one, up to a maximum of 3. The maximum score in the knowledge section was 15.

### 2.2. Statistical Analysis

The questionnaire data was analysed in Minitab Version 16. The demographic background of participants was matched to the categories of the most recent Queensland Census 2011. Knowledge scores (K score) were determined for each respondent by the total number of correct answers out of 15 questions. This assumes that each question was the most relevant to test information on this aspect of the production system and contributes equally to a respondent’s total knowledge. Numerical distribution of the K scores was examined and, to determine its influence on attitude and consumption, the total score was regressed against 21 predictors describing attitude and consumer behaviour using forwards backwards stepwise regression with alpha levels of 0.015 and fitted intercepts. Effects of the predictors found to be significantly (*p* ≤ 0.05) correlated with knowledge scores were entered into a General Linear Model to examine the differences between levels. For this purpose, Knowledge scores were transformed to square root to approximate a normal distribution of residuals. Both back-transformed and untransformed means are provided. Pairwise comparisons were carried out using Tukey’s test.

Logistic regression analyses (either binary, nominal or ordinal, as appropriate to the response structure) were used to analyse the effects of demographic variables and knowledge scores on the attitude and behaviour questions. For example, to evaluate whether place of residence influenced responses to attitude questions, urban residents were used as the referent base group and compared to the other three groups, acreage/large blocks, rural (country town) and rural (farming property) using nominal logistic regression. Referent base groups were selected as those with the most responses, except that males were used rather than females since either group can be chosen without affecting the analysis if there are only two. A principal component analysis was used to cluster responses to attitude (13 questions) and knowledge (15 questions) questions.

## 3. Results

Of the 2663 eligible participants approached, 506 answered the survey, a response rate of 19%. The average response time was estimated at 18 min. There were 205 males and 286 females, a higher female proportion compared with Queensland census data (Table 1). Fifteen respondents chose not to disclose their gender. The most common age bracket was 30–49 (50.7%), and the most common education level was to college or university degree level. Our respondents were more numerous in the 40–49 year old category and less numerous in the 60 and over category compared with the Queensland census data of 2011, and represented a more educated sample of the population. Most were urban dwellers (87%), with few rural town dwellers (6%) or acreage (large block) dwellers (5%). Respondents’ income status was similar to the Australian average of AUD $78,000/year [26]. Most (*n* = 305, 64%) were partnered with children, more than in the state of Queensland, and almost half (*n* = 234, 46%) were of the Christian religion, fewer than in the state.

### 3.1. Respondents’ Knowledge

Respondents’ level of understanding of chicken production systems was most commonly reported as no, little or some knowledge, with fewer than 10% responding that their knowledge was good or expert (Table 2). Most gained their knowledge from the Internet and media, with a significant number gaining it from friends. Most had never visited a chicken farm, and, of the approximately one-third that had visited one before, it was not recent for most.

**Figure 1:**
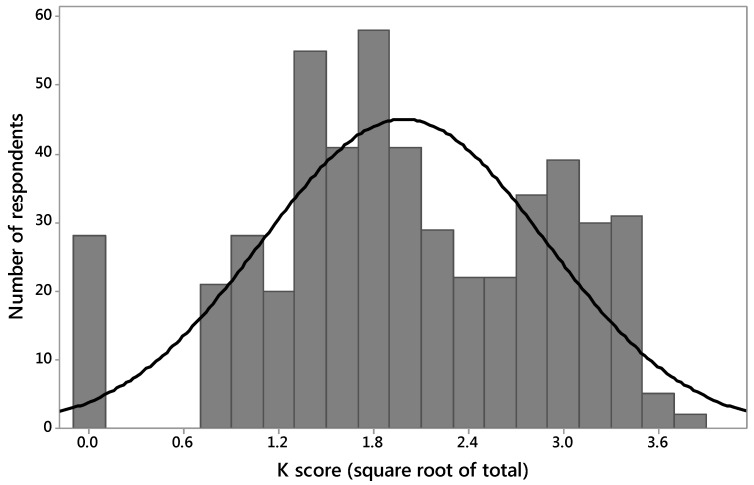
Distribution of K scores (out of 15) approximated a normal distribution curve with a higher than expected number of zero values (*n* = 28). The mean value was 1.99 (K score 3.96/15), Standard Deviation 1.24, and Median Value 2.00 (K score 4/15).

The distribution of K scores was not normal, but √K score approximated a normal distribution, except that there was a higher than expected number of zero values (*n* = 28) (Figure 1). The mean value for √K score was 1.99 (K score 3.96/15), median 2.0 (K score 4.0/15), with a Standard Deviation of 1.24. Given that the mean and medians were very similar, √K score values were used for analysis.

After eight knowledge questions (Appendix A), respondents were asked how long it takes for intensively-reared meat chickens to reach a slaughter weight of 2 kg. Only 47 respondents (9%) gave the correct answer of 35–45 days, 32 (6%) said 30–35 or 45–50 days and 427 (84%) gave answers outside of these choices. Respondents were asked, what are three of the biggest welfare problems for meat chickens in barns? A total of 156 respondents (31%) gave three valid responses, a further 65 respondents (13%) gave two valid responses and a further 48 respondents (9%) gave one valid response. The remaining 47% (*n* = 237) did not respond. The most common responses were poor lighting systems, too little space per bird, unable to reach feeders, unable to spread wings and too rapid growth. When the same question was asked for chickens in transport, 143 respondents (28%) gave three valid responses, a further 63 respondents (12%) gave two responses and a further 50 respondents (10%) gave one response. The most common responses were overcrowding, hot temperatures, odour, absence of food and water, and long distances.

### 3.2. Attitudes towards Welfare in Chicken Rearing System

**Figure 2:**
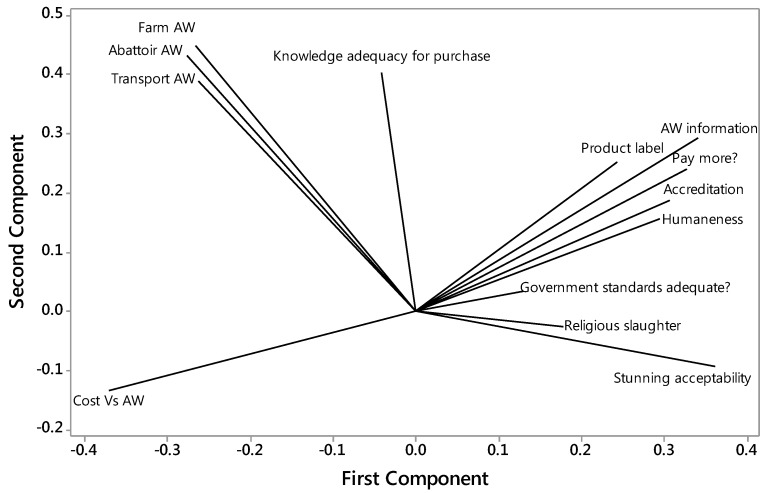
Biplot of Principal Component Analysis of attitude questions, showing the first two components. The first component appears to relate to purchasing issues and the second to pragmatic issues of providing for animal welfare. AW = animal welfare.

A cluster analysis of the attitude questions produced 4 components with eigen values > 1, explaining 61% of the variation in total. A biplot of the first two components demonstrated that there were similar responses to questions about how good or bad animal welfare was on the farm, during transport and in the abattoir (Figure 2). Similar responses were observed at the opposite end of the scale for the first component for questions relating to attitudes to consumption, and to the two questions about chickens being conscious (religious slaughter and stunning acceptability). The first component appears to relate to purchasing issues, with Cost versus Animal Welfare (the cost of chicken meat is more important to me than the chicken’s welfare) at one end and willingness to pay more, including of accredited products, at the other end. The second component appears to relate to providing for animal welfare (most positive) versus pragmatic issues of cost and religious concerns (least positive).

Most respondents were unsure whether chickens reared in meat production systems are protected by government standards which ensure that the welfare of birds is adequate (Table 3). They were also either unsure what they thought about meat chicken welfare on farms and during transport, or they thought it was good, bad or neither good nor bad in approximately equal numbers. Very few thought it was very good or very bad. In the abattoir (Table 4) most were unsure, but many thought it was neither good nor bad and a significant proportion (17%) thought that it was good.

Most respondents (54%) felt that it was unacceptable or very unacceptable that 1% of birds do not get adequately stunned by normal abattoir practices (Table 4). A similar proportion (58%) felt that it was unacceptable that some Australian abattoirs are allowed to kill chickens that are conscious, for religious reasons (Table 4). Most respondents (83%) agreed or strongly agreed with the statement “Food must be produced and processed from chickens that are treated humanely” (Table 3).

There was no consensus among respondents about whether their knowledge of the welfare of meat chickens was sufficient to allow them to form an opinion about which chicken products they should purchase (Table 4), but 49% disagreed or strongly disagreed that the cost of chicken meat was more important to them than the chicken’s welfare, compared with only 27% agreeing or strongly agreeing (Table 3).

### 3.3. Consumption and Attitudes towards Labelling

The most common chicken products purchased were chicken pieces or whole chicken, not flavoured or processed products (Table 3). Free range chicken was the most common branded product purchased, followed by whole chicken and chicken portions (Table 3). Chicken was most commonly eaten weekly, and if not it was most likely to be eaten less than once a week (Table 4). Most bought it fresh (61.0%), not frozen (Table 4). Most respondents considered labelling of production systems important (Table 4), and an overwhelming majority (63%) wanted to see information regarding welfare wherever chicken products are sold (Table 3) and were specifically looking to buy accredited chicken products (Table 4). Just over half (56%) said that they were prepared to pay to set up animal welfare ratings but most commonly at the lowest option, $0.50 (AUD) per product item (Table 3), although some (14%) were willing to pay whatever it costs.

### 3.4. Relationships between Respondents’ Knowledge and:

#### 3.4.1. Demographics

Respondents’ K scores increased with age from 1.9/15 for respondents ≤19 years to 5.5/15 for respondents aged 50–59 (Table 5). College certificate or diploma graduates had higher levels of knowledge than either respondents with high school certificates or university graduates. Acreage dwellers had higher knowledge scores than urban, rural town and other dwellers. K score was greatest for single people with no children.

#### 3.4.2. Attitudes

When asked about the welfare of meat chickens at Australian abattoirs, respondents who rated it very bad or very good had higher K scores than those with intermediate ratings (Table 4). Those who were unsure had the lowest K score. When told that 1% of birds do not get adequately stunned by normal abattoir practices prior to slaughter, respondents who regarded the practice as unacceptable had high K scores of 4.58 compared with respondents with no strong feelings regarding the issue with a K score of 4.93. When asked if their knowledge about the welfare of meat chickens was sufficient to allow them to form an opinion about which chicken products to purchase, respondents who agreed had higher K scores than those who disagreed.

#### 3.4.3. Consumption

Respondents’ K scores increased with the frequency of eating chicken, from <1/week to daily. However, those who did not eat chicken because they were vegetarian or they did not like chicken had intermediate K scores, lower than those with the highest consumption rate. Frozen chicken purchasers tended to have higher K scores than consumers who bought fresh products or a mixture of fresh and frozen (Table 4). Consumers who were willing to buy products with accredited labelling had lower K scores than those that were not. When told that some Australian abattoirs are allowed to kill chickens without them being unconscious for religious reasons, respondents who rated the practice as very unacceptable had a low K score of 2.99 (Table 5), compared with other acceptability ratings.

### 3.5. Relationships between K Score and Attitude/Consumption

In the stepwise regression, K scores were regressed against 21 predictors about attitudes to chicken meat production system, consumption of chicken and demographics (Appendix B). The final model included 13 significant predictors and had an R^2^ of 43%. The most important predictor was that as people said they had a greater understanding of chicken production systems, their K score increased. The second most important predictor was that high K scores were closely correlated with a self-reported low level of education, and the third most important predictor was that it was acceptable to kill chickens without stunning for religious purposes. Those with high K scores were more likely to be older, single with no children and agreeing that their knowledge is sufficient to form an opinion when purchasing chicken products. Of next importance was that they ate chicken frequently, they purchased frozen products and that they did not purchase chicken products with accredited labelling. They also regarded the welfare of meat chickens at the abattoir as good, and they were more likely to live in rural areas.

### 3.6. Gender Effects

#### 3.6.1. Knowledge

For most questions females had the same level of understanding as males, however females were more likely to incorrectly identify food fed to chickens as not being of vegetable origin and more males than females thought that chickens’ diets would include grass and hay (Appendix C).

#### 3.6.2. Attitudes

More females than males thought that the welfare of meat chickens at Australian abattoirs was bad (Table 6). Females considered it less acceptable than males that approximately 1% of chickens do not get adequately stunned at the abattoir and that some Australian abattoirs are allowed to kill chickens without them being unconscious for religious reasons. More females than males agreed with the statement “Food must be produced and processed from chickens that are treated humanely.”

#### 3.6.3. Consumption

Females were less likely than males to regard the cost as more important than welfare in chicken production. Females were more likely than males to buy free range chicken products (143 compared to 70), whole chicken (108 compared to 79) and processed chicken products, e.g., chicken schnitzel (57 compared to 15). Females said that they ate chicken less frequently than males. Males were more interested than females in seeing information regarding the welfare of chickens at the point of sale and were more prepared to contribute to the cost of setting up animal welfare ratings on animal products, by paying extra for the product.

### 3.7. Place of Residence Effects

#### 3.7.1. Knowledge

Compared to acreage dwellers, urban dwellers thought that birds in barns had greater space availability (Table 7). Participants living on acreage were more likely to be incorrect in questions about housing, gender determination and stunning, compared to urban dwellers. Rural dwellers were more likely to be correct in relation to gender determination, but incorrect in relation to housing systems, they were also more likely to believe that chickens travelled further to the abattoir, compared to urban dwellers.

#### 3.7.2. Attitudes

Acreage dwellers more strongly agreed than urban dwellers that the welfare of chickens reared for meat production systems is inadequately protected by government standards to ensure the welfare of the birds. Urban respondents were more likely to believe that the welfare of meat chickens on the farm is bad. Acreage dwellers found the facts that 1% of birds are not adequately stunned and abattoirs slaughter birds without stunning for religious reasons more acceptable than did urban dwellers. They more strongly disagreed that chickens must be treated humanely, and agreed that their meat chicken welfare knowledge is adequate to form an opinion about purchases, compared with urban dwellers. They agreed more than urban dwellers that the cost of chicken was more important than chicken welfare.

#### 3.7.3. Consumption/Labelling

Acreage and rural dwellers said that they were likely to eat chicken more often than urban dwellers. Rural dwellers considered product labelling less important than urban dwellers, and both acreage and rural dwellers were less interested in seeing information regarding the welfare of chickens at the point of sale or to seek to purchase chicken products with accredited labelling systems.

### 3.8. Marital Status Effects

#### 3.8.1. Knowledge

Single respondents with no children thought that they had more limited understanding of chicken production systems than those who were partnered with no children, and were most likely to be correct for three questions (Table 8).

#### 3.8.2. Attitudes

Respondents who were single with no children were more likely than partnered respondents with no children to agree that the welfare of meat chickens is adequately protected by government standards. Widowers rated meat chicken welfare on the farm and in the abattoir to be worse and during transport to be better than single respondents without children; they also were more accepting of inadequate stunning procedures than single respondents without children. Partnered respondents with children rated welfare during transport to be better as well but single respondents with children rated it worse. Partnered respondents without children rated welfare worse in the abattoir. Partnered respondents with no children found killing without stunning for religious reasons more acceptable than widowers and single respondents without children. Single respondents without children agreed more with the statement that food must be produced and processed from chickens that are treated humanely than single or partnered respondents with children.

#### 3.8.3. Consumption

Single respondents with children agreed more than widowers that their welfare knowledge is sufficient for chicken product purchase, and they considered labelling information about chicken farming systems more important than did partnered respondents with children. They wanted information on welfare of chickens at point of sale more than any other group, and they, and widowers, were more likely than those without children to say that they would pay for the cost of setting up animal welfare ratings on products.

### 3.9. Religion Effects

#### 3.9.1. Attitudes

Muslims thought that the fact that 1% of birds are not adequately stunned was more acceptable than Christians (Table 9). Jews and atheists found it less acceptable than did Christians. Muslims also found it much more acceptable to kill chickens without stunning for religious reasons than did Christians. Christians more than Muslims, Jews and atheists agreed with the statements that food must be from chickens that are treated humanely and that cost was more important than the chicken’s welfare more than Muslims, Jews and atheists. Compared to Muslims, atheists and Buddhists, Christians more strongly believed that their welfare knowledge about meat chickens was not sufficient for food purchasing, compared to Muslims, atheists and Buddhists.

#### 3.9.2. Consumption/Labelling

Christians thought that product labels giving details of chicken rearing systems were more important when making purchases than did Muslims and atheists.

### 3.10. Age Effects

As age increased, respondents were willing to pay less for an animal welfare rating (Table 10); they were more likely to select products with a Heart Foundation approval and more likely to choose chicken portions or corn/whole grain-fed chickens.

### 3.11. Income Effects

As income increased, respondents were more likely to believe that the welfare of meat chickens on the farm (Regression Coefficient 0.16, OR 1.18, *p* = 0.04) and during transport (Regression Coefficient 0.16, OR 1.18, *p* = 0.04) was bad. They were also more likely to believe that chickens can be killed for religious reasons without stunning (Regression Coefficient 0.24, OR 1.27, *p* = 0.002) and to know that gender could be determined from feathers (Regression Coefficient −0.28, OR 0.75, *p* = 0.02) and to know the distance that chickens travelled to the abattoir (Regression Coefficient 0.35, OR 1.42, *p* < 0.0001). However, they were less likely to know that birds are usually stunned before slaughter (Regression Coefficient −0.32, OR 0.73, *p* < 0.0001).

## 4. Discussion

The response rate of 19% was similar to other farm animal welfare surveys [21]. Randomly approaching members of the public who were not aware of the nature of the survey helped to minimize any potential bias [28,29]. However, some selection bias is evident and in particular the higher education level of the respondents compared to the Australian population could potentially influence people’s understanding of chicken production systems. The preponderance of middle-aged respondents, compared with the Australian population, may have influenced our results on consumption (Table 9) and knowledge scores (Table 4). Most respondents indicated that they were urban dwellers, which is representative of the Australian population. Gaining contemporary knowledge about the industry was through the internet, journals, newspaper articles, television programmes and more noticeably, through friends and acquaintances. Further work on knowledge sources is warranted as the Australian public spends about $5.6 billion per year on poultry products [9].

### 4.1. Knowledge

K scores generally increased with self-rated knowledge of chicken welfare, adding validity that objective knowledge matched subjective knowledge assessment. The disproportionately high number of zero values in the knowledge score suggests that some respondents deliberately avoided answering all knowledge questions, but this may also have been because they genuinely did not know the answers. The majority of urban respondents (87%), compared with the Australian average of 63% [30], would be less likely than rural dwellers to be familiar with farming systems, which could contribute to low knowledge scores.

The knowledge questions demonstrated that public knowledge of chicken production systems was limited, with many participants possessing little or no knowledge of the industry and a median knowledge score of 4 out of 15, indicating that they answered four questions correctly out of 15, i.e., 27% (and mean of 3.96/15, Section 3.1). Bergman and Maller [20] studied the factors leading Australians to support or reject factory farming, especially poultry and pig productions, and concluded that Australian consumers knew little about these systems, there was significant confusion and scepticism about ‘organic’ & ‘free range’ labelling and limited trust in the RSPCA labelling systems. Napolitano et al. [18] examined the effect of information about animal welfare, expressed in terms of rearing conditions, on acceptability of lamb for consumption. Prior knowledge of rearing conditions influenced their perceived acceptability, with worse scores given to meat if they knew it had been reared artificially, rather than by their mother. According to Costell et al. [21], the hedonic acceptability of food items is related to whether our perception of food differs from the expected, which in turn may be influenced by understanding of production and processing systems involved in producing the food item.

Some responses indicated that chicken welfare problems become a banality as K score increases, for instance believing that it was acceptable to kill chickens without stunning for religious purposes. Although increasing K score was associated with increased self-reported knowledge of chicken meat production systems, it was associated with low levels of general education. The latter may indicate that high K score respondents lacked the broad education necessary to empathize with chickens in poor welfare conditions.

### 4.2. Attitudes

The attitude of 52% of respondents was that meat chicken welfare on the farm was neither good nor bad, or they were unsure, suggesting that there is a great deal of uncertainty about this issue. Similarly, 49 and 62% of respondents had no definite attitude regarding the welfare of meat chickens during transport or at the abattoir, respectively. By contrast, in Europe most (77%) of the public believe that improvement in animal welfare is needed, with meat chickens being one of the systems of production most in need of reform [31]. Similarly, there was little agreement about whether the existing Australian standards ensured that the welfare of reared meat chickens is adequate. Mench [32] and Sumner et al. [33] suggested that standards should not only minimise animal suffering during transport and slaughter but maintain quality of life for animals throughout their production life. Such uncertainty appeared to link to respondents’ lack of knowledge, with those that were unsure about the chickens’ welfare in abattoirs having lowest knowledge scores. Similarly those that had no strong feelings in relation to inadequate stunning, which may indicate uncertainty, also had low scores. Lowest scores in this question were given by those finding it very acceptable, giving some credence to a relationship between empathy towards the chicken and knowledge.

### 4.3. Consumption

With the exception of those that avoided chicken because they were vegetarian or they did not like it, K score increased considerably with the frequency of consumption, and more knowledgeable respondents were less likely to buy products with accredited labelling. The latter may be explained by those with knowledge believing accreditation to be unnecessary for their choice of chicken product. Regarding the frequency of chicken consumption, one possibility is that people consuming more chicken are interested to learn about the industry. Another is that people of higher socioeconomic status were more knowledgeable about farming systems and ate more chicken because they are more aware of its health benefits. However, the more knowledgeable respondents were less willing to pay for accredited labelling for chicken welfare, which would not be expected of high socioeconomic respondents. A third possibility is that the more knowledgeable, frequent chicken consumers were connected with the industry, however, we considered this unlikely as only 1% lived on a chicken farm, 5% had visited one in the last two years and 6% indicated that they had gained their knowledge as farm employees.

The type of chicken meat that respondents said they were most likely to buy was free range chicken products, whereas Australian free range chicken meat production accounts for only 10 to 15% of the total production [34]. This properly reflected an intention or desire, rather than actuality. Furthermore, a total of 63% of respondents sought to purchase a chicken product with accreditation, particularly if they had little knowledge about chicken production systems. This suggests that consumers are using accreditation as a means of ensuring products are of high welfare, replacing their limited knowledge, even though accredited labels exhibit no information regarding the conditions where birds were raised or processed and no reference to animal welfare [35]. Consumers have put pressure on retailers to properly label products and on producers, manufacturers and supermarkets to have an animal welfare labelling system [23] as well as the country of origin, production techniques [36] and conditions of rearing [19]. Fifty-six percent of respondents were prepared to contribute to the cost of setting up animal welfare ratings by paying extra for the products, particularly females, and the most common increase in cost that would be accepted was 2.5%. A study in Chile indicated a willingness to pay up to 15% more for meat produced to improved animal welfare standards [19]. European consumers have indicated their willingness to change their usual place of shopping to be able to purchase more animal-welfare-friendly products [37]. Consumers are also willing to pay more for natural or organic chicken [23], with the latter being perceived as safer, healthier and having fewer pesticides, hormones and antibiotics than other meat [38]. Labelling systems are based on transparency, informing consumers that the products have satisfied the welfare conditions where animals were reared, transported and processed [39].

### 4.4. Demographic Effects

On ethical issues, socio-demographics as explanatory variables of behaviour may be less influential than values, attitudes, motives and lifestyles. In our study we had major effects of gender and dwelling place on attitudes and consumption, whereas religion had the most influence on attitudes but little on consumption.

#### 4.4.1. Gender

There was no evidence that females had a better understanding of the chicken production system than males. However, it is recognised elsewhere that females have greater knowledge of animal welfare concerns, with males being more traditional in their purchasing habits for animal products [40]. Females displayed greater sensitivity to chicken welfare than males, confirming much previous research [41,42]. Females were more ethical about their chicken consumption intentions, and reported being twice as likely to buy free range but only slightly more likely to buy whole chicken. They were also much more likely to buy processed products, which may reflect their role in managing the nutrition of children. They reported buying less chicken than males, confirming a Eurasian survey which found that female students reported that they ate poultry less commonly than male students [43]. Males’ showed greater interest than females in seeing information regarding the welfare of chicken at the point of sale than females and even being more prepared to pay for this conflicts with other studies [44,45] which found that females were willing to pay extra for certified food products. Females reporting less frequent consumption of chicken than did males probably reflects the fact that women show more health-related behaviours and considered attitudes towards food than men [46,47].

#### 4.4.2. Place of Residence

Dwellers on acreage/large blocks were more knowledgeable than most other groups, but they were generally less sympathetic to chicken welfare than urban dwellers, in relation to stunning practices and treating birds humanely. The acreage or large block dwellers are more likely to keep chickens and gain their attitudes towards chicken welfare from this practice, rather than through the media, which would be the case for urban dwellers. Acreage/large block dwellers also ate more chicken than urban dwellers and were less interested in labelling about chicken welfare, even though they thought government standards were less than adequate to protect welfare. The latter suggests a better knowledge but less concern in acreage/large block dwellers, compared with urban dwellers.

#### 4.4.3. Marital Status

Single respondents with children were most likely to want information on the welfare of chicken at point of sale, probably reflecting their limited time for shopping, and they considered this information more than some other groups. Other research has identified that single parents with children spend more of their food budget eating away from the home, compared to partnered respondents with children [48]. The study suggested that the most sympathetic consumers were single respondents without children, as they rated welfare worse on the farm and in the abattoir and agreed most that chickens must be treated humanely. They were also least accepting of inadequate stunning or avoiding stunning for religious reasons. Results for widowers should be treated with caution as they are confounded with age.

#### 4.4.4. Religion

Muslims knew more about stunning than Christians and they were less likely to find it acceptable, reflecting their belief that animals must be alive when their throats are cut and must die from loss of blood [49]. Overall 54% of our respondents’ believed that the practice of slaughtering birds without adequate stunning was unacceptable, probably because of the welfare impact [50]. There was an apparent contradiction between Christians having greater regard for cost than an animal’s welfare but also requiring chicken to be from animals that are treated humanely, compared to Muslims, Buddhists and atheists.

#### 4.4.5. Age, Income and Education

The reduced willingness to pay for animal welfare ratings as respondents aged may reflect reduced disposable cash for this purpose, or it may reflect changing attitudes, this not being a longitudinal study. Greater tolerance to not stunning the chicken for religious reasons was evident in higher income respondents, confirming previous findings in Chinese studies [43]. A greater willingness to recognise poor welfare on farm and during transport in high income respondents may reflect a greater ability to pay for high welfare products. Respondents with a low level of education had high K scores. This suggests that there was a cohort of poorly-educated respondents who had knowledge of the poultry industry.

## 5. Conclusions

Public knowledge of the Australian poultry production systems was limited. Most was indirectly gained from the media, and few respondents had direct experience with chicken farming. Our finding that knowledge related to an improved attitude towards chicken welfare is valuable, since it suggests that informing the public about chicken welfare could increase levels of concern. However, this was not associated with increased consumption of high-welfare products; in fact, high-level consumers had a natural suspicion of accreditation programmes that would make it difficult to improve animal welfare through this method. The observed positive relationship between chicken consumption and knowledge may derive from a belief in respondents who ate relatively more chicken that they should understand the systems of production of the animals that they are consuming. The connection between knowledge and attitudes suggests that educating consumers might help to improve their empathy towards meat chickens, but the lack of relationship between empathy and consumption and the suspicion of accreditation systems suggests that any increased empathy will not necessarily have an impact on the sales of high-welfare products.

More scientific studies are needed to support public demand for improving the welfare conditions of chickens, as they were at least willing to contribute a small amount (median about 5%) to establish labelling systems that take into account the welfare of birds. The study also identified those consumers who were most concerned about the welfare of chickens in this context: females, urban dwellers and relatively high-income respondents.

## Figures and Tables

**Table 1:** Demographics of respondents compared with data from Queensland, Australia (*n* = 506).

		Number of Respondents	% of Survey Sample	Queensland Data, % *
Gender	Male	205	41.7	49.6
	Female	286	58.2	50.4
Age	18–19	36	7.4	27.0 **
	20–29	66	13.6	13.7
	30–39	111	22.9	13.7
	40–49	135	27.8	14.2
	50–59	98	20.2	12.7
	60 & over	39	8.0	18.7
Education	No formal schooling	0	0	0
	Primary	10	2.06	29.7
	Secondary	74	15.2	20.2
	Technical College	61	12.5	6.2
	University	184	37.9	13.5
	Higher University Degree	141	29.0	30.4
	Other	16	3.3	
Dwelling	Urban	421	86.6	
	Acreage	26	5.3	
	Rural–town	27	5.6	
	Rural–farm	9	1.8	
	Other	3	0.62	
Annual Income	Less than $20,000	95	22.3	Mean $78,000
	$20,000–$39,000	47	11.0	
	$40,000–$59,000	75	17.6	
	$60,000–$80,000	81	19.0	
	>$80,000	128	30	
Marital Status	Single, no children	100	20.9	39.2
	Single, with children	20	4.2	7.8
	Married/De Facto	43	9.0	6.0
	no children			
	Married/De Facto	305	63.7	42.0
	with children			
	Widowed	11	2.3	5.0
Religion	Christian	234	46.1	64.8
	Jewish	12	2.4	0.1
	Hindu	1	0.2	0.7
	Buddhist	7	1.4	1.5
	Muslim	22	4.3	0.8
	Atheist	53	10.5	22.1
	Other	36	7.1	10.0 ***
	No response	141	27.9	

* [26,27]; ** [26] lists only 15–19 years of age; *** Includes other religions and/or not stated.

**Table 2:** Number and % of respondents with answers to knowledge questions that were not significantly (*p* < 0.05) related to respondents’ knowledge (K score).

Questions and Response Options	Number of Respondents	% of Respondents
**Knowledge of chicken production systems**		
Self-rated understanding of chicken production system		
Expert	7	1.4
Good knowledge	38	7.5
Some knowledge	134	26.5
Little knowledge	191	37.7
No knowledge	136	26.9
Source of knowledge		
Formal qualifications—relevant degree, training course	15	3.7
Farm employment—hands-on experience, relevant training course	23	5.7
Personal interest, e.g., internet, journals, newspaper articles, television programmes	223	55.1
Friends and acquaintances	136	33.6
All of the above	8	2.0
Visits to a chicken production farm?		
Yes, in the last two years	25	4.9
Yes, more than two years ago or on a school trip	153	30.2
I live on a chicken production farm	4	0.8
Never	324	64.1

**Table 3:** Number and % of respondents in each category for attitudinal and consumption questions, for questions that were not significantly (*p* < 0.05) related to respondents’ knowledge (K score).

Questions and Response Options	Number of Respondents	% of Respondents
**Attitudes regarding chicken rearing systems**		
Australian meat chickens are not protected by government welfare standards		
Strongly agree	32	6.5
Agree	84	16.9
Neither agree nor disagree	233	47.0
Disagree	137	27.6
Strongly disagree	10	2.0
Welfare of Australian meat chickens on the farm		
Very good	23	4.6
Good	105	20.8
Neither good nor bad	120	23.8
Bad	99	19.6
Very bad	17	3.4
Unsure	141	27.9
Welfare of Australian meat chickens during transport		
Very good	15	3.0
Good	108	21.3
Neither good nor bad	101	20.0
Bad	89	17.6
Very bad	44	8.7
Unsure	149	29.5
Killing chickens that are conscious for religious reasons in Australian abattoirs		
Very unacceptable	173	36.1
Unacceptable	106	22.1
No strong feelings	89	18.5
Acceptable but with some reservations	80	16.7
Perfectly acceptable	32	6.7
Food must be produced and processed from chickens that are treated humanely		
Strongly agree	250	51.4
Agree	155	31.9
Neither agree nor disagree	64	13.2
Disagree	15	3.1
Strongly disagree	2	0.41
Cost of chicken meat is more important to me than the chicken’s welfare		
Strongly agree	32	6.6
Agree	101	20.7
Neither agree nor disagree	114	23.4
Disagree	166	34.1
Strongly disagree	74	15.2
**Consumption of chicken products**		
What brands of chicken meat are you most likely to buy?		
Free range	213	42.1
Corn or whole grain fed	46	9.1
Cheapest/home brand/on special	95	18.8
Products from a known producer	71	14.0
Products with heart foundation tick	41	8.10
Whole chicken	187	37.0
Chicken portions	177	35.0
Processed chicken products	72	14.2
What type of chicken products do you usually buy?		
Whole chicken	275	54.4
Chicken pieces	343	67.8
Flavoured chicken meals	72	14.2
Processed chicken meat	84	16.6
Importance of rearing system on the product label when purchasing chicken products		
Very important	144	29.3
Quite important	164	33.3
Neither important nor unimportant	97	19.7
Not very important	64	13.01
Not important at all	23	4.7
Need for chicken welfare information wherever they are sold?		
Yes	308	63.1
No	82	16.8
Not interested	98	20.1
Amount you would be willing to pay to set up animal welfare ratings on animal products		
50 c/product if cost is ≤ $20	118	45.5
$1.00/product if cost is ≤ $20	42	16.2
$2.00/product if cost is ≤ $20	19	7.3
Whatever it costs to include	37	14.3
Should be done but I shouldn’t pay	43	16.6

**Table 4:** Number and % of respondents in each category for attitudinal and consumption questions, for those questions with significant relationship to knowledge (K) score, together with the K score for responders to each option and probability of these being different (Standard Error of the Difference between any two √K score means = 0.042).

Questions and Response Options	Number of Respondents	% of Respondents	√K Score	K Score/15
**Attitudes**				
Welfare of Australian meat chickens at the abattoir				
Very good	8	1.6	2.34 ^a^	5.47
Good	87	17.2	1.92 ^ab^	3.69
Neither good nor bad	141	27.9	1.87 ^b^	3.50
Bad	62	12.3	2.13 ^ab^	4.54
Very bad	34	6.7	2.35 ^a^	5.52
Unsure	174	34.4	1.57 ^c^	2.46
*p* value			0.001	
1% of birds do not get adequately stunned in abattoir practices				
Very unacceptable	92	19.3	2.99 ^a^	6.15
Unacceptable	164	34.5	2.20 ^a^	4.84
No strong feelings	130	27.3	1.74 ^b^	3.03
Acceptable with reservation	70	14.7	2.21 ^a^	4.88
Very acceptable	20	4.2	1.52 ^b^	2.31
*p* value			0.001	
Killing chickens that are conscious for religious reasons in Australian abattoirs				
Very unacceptable	173	36.0	1.73 ^b^	2.99
Unacceptable	106	22.1	2.14 ^a^	4.58
No strong feelings	89	18.5	2.22 ^a^	4.93
Acceptable with reservation	80	16.7	1.93 ^ab^	3.72
Very acceptable	32	6.7	2.13 ^ab^	4.53
*p* value			0.007	
Self-rated knowledge of chicken welfare is enough to form opinion about buying chicken products				
Strongly agree	35	7.3	2.14 ^ab^	4.58
Agree	138	28.6	2.39 ^a^	5.71
Disagree	91	18.9	2.04 ^b^	4.16
Strongly disagree	37	7.7	1.73 ^b^	2.99
*p* value			0.001	
**Consumption/labelling**				
Number of times per week you eat chicken				
Never/I’m vegetarian	7	1.5	2.32 ^bc^	5.38
Never/Don’t like chicken	21	4.4	1.79 ^bcd^	3.2
<1/Week	133	28.0	0.66 ^d^	0.43
Once/Week	299	63.0	1.74 ^c^	3.03
2 or 3/Week	11	2.3	2.14 ^b^	4.58
Daily	4	0.8	3.53 ^a^	12.46
*p* value			0.001	
Type of chicken meat consumers buy				
Fresh	288	60.8	1.93 ^ab^	3.72
Frozen	37	8.8	2.26 ^a^	5.11
Mix of Both	149	31.4	1.90 ^b^	3.61
*p* value			0.05	
Labelling—would you purchase a product with accredited labelling?				
Yes	307	*63.2*	1.91	3.65
No	179	*36.8*	2.15	4.62
*p* value			0.002	

Means with different superscripts differ significantly (*p* < 0.05) by the Tukey’s test. √K Score= square root of the K score.

**Table 5:** Number and % of respondents to questions with significant relationship to knowledge (K) score, together with the K score for responders to each option (Standard Error of the Difference between two means = 0.042) and probability of these being different.

Questions and Response Options	√K Score	K Score/15
**Demographics**		
Age		
≤19	1.39 ^c^	1.93
20–29	1.97 ^ab^	3.88
30–39	1.99 ^b^	3.96
40–49	2.30 ^a^	5.29
50–59	2.35 ^a^	5.52
≥60	2.18 ^ab^	4.75
*p* value	<0.001	
Highest level of education		
Primary	1.45 ^abc^	2.10
High school	2.20 ^b^	4.84
Technical college certificate/diploma	2.73 ^a^	7.45
College/university degree	2.17 ^b^	5.88
Higher university degree	2.31 ^b^	5.34
Other	1.32 ^c^	1.74
*p* value	0.001	
Place of residence		
Urban—city/town	2.06 ^b^	2.24
Acreage/large block	2.61 ^a^	6.81
Rural—country town	1.88 ^b^	3.5
Rural—farming property	2.06 ^ab^	4.24
Other	1.45 ^b^	2.10
*p* value	0.002	
Marital status		
Single, no children	2.30 ^a^	5.29
Single, children	1.82 ^ab^	3.31
Partnered/de facto, no children	2.13 ^ab^	4.53
Partnered/de facto, children	1.83 ^b^	3.35
Widowed	2.08 ^ab^	4.32
*p* value	0.001	

Means with different superscripts differ significantly (*p* < 0.05) by the Tukey’s test.

**Table 6:** Significant differences in attitudes and consumer behaviour between the gender groups. Mean values are shown for the referent group for gender, male respondents, and the comparative group, female respondents, as well as Odds Ratio and *p* value for the difference.

Questions and Response Options	Males	Females	Coefficient	Odds Ratio	*p* Value
**Attitudes**					
Chicken welfare at the abattoir, 1 vg–5 vb	2.80	3.17	−0.79	0.45	0.001
1% of birds do not get adequately stunned in abattoir practices, 1 vu–5 va	2.66	2.38	0.57	1.77	0.007
Abattoirs slaughter birds without stunning, 1 vu–5 va	2.49	2.26	0.91	2.49	0.001
Chicken must be treated humanely, 1 sa–5 sd	1.78	1.63	0.63	1.87	0.007
Cost of chicken is more important than chicken’s welfare, 1 sa–5 sd	3.09	3.46	−0.74	0.48	0.001
**Consumption/labelling**					
What chicken products do you buy?					
Free Range (no. respondents)	70	143	0.90	2.46	0.001
Processed (no. respondents)	15	57	−1.11	0.33	0.003
Whole (no. respondents)	79	108	−0.53	0.59	0.004
Chicken consumption (1 never, 6 daily).	3.67	3.62	0.699	2.01	0.006
Need information on chicken welfare (1 yes, 2 dk, 3 no).	1.60	1.49	0.53	1.69	0.04
Willing to pay more for animal welfare (1 yes, 2 no).	1.49	1.41	−0.43	0.65	0.04

vg = very good, vb = very bad, vu = very unacceptable, va = very acceptable, sa = strongly agree, sd = strongly disagree, dk = don’t know.

**Table 7:** The difference in attitudes towards meat chicken welfare according to dwelling place: urban (city/town) (referent group, 1), acreage/large block (AC) (group 2), rural (country town) (group 3), rural (farming property) (group 4) and other dwellers (group 5).

Questions and Response Options	Base Line Group	Comparative Group	Coefficient	Odds Ratio	*p* Value
**Knowledge**					
Space for each bird in barn, (1, 0.25 m^2^–4, 5 m^2^)	1: 1.61	2: 1.34	2.08	8.00	0.003
Housing the same for egg and meat production, 1 T, 2 DK, 3 F	1: 2.14	2: 2.64	−2.06	0.13	0.000
		3: 2.32	−2.52	0.08	0.003
Feather sexing of chicken, 1 T, 2 DK, 3 F	1: 2.17	2: 2.41	−0.94	0.39	0.47
		3: 1.69	2.24	9.43	0.001
Chicken travelling distance to abattoir	1: 2.23	3: 2.39	−1.46	0.23	0.02
Normal practice for meat chickens to be stunned before slaughter? (1 yes, 2 Dk, 3 No)	1: 1.92	2: 3.19	−2.37	0.09	0.001
**Attitudes**					
Chicken welfare not adequately protected by government standards, 1 sa–5 sda	1: 2.96	2: 3.38	−0.89	0.41	0.038
Chicken welfare on farm, 1 vg–5 vb	1: 2.99	2: 2.69	1.48	4.34	0.001
Unstunned birds at abattoir, 1 vu–5 spa	1: 2.44	2: 3.19	−1.51	0.22	0.001
Abattoirs slaughter birds without stunning, 1 vu, 5 pa	1: 2.34	2: 2.92	−1.11	0.33	0.007
Chicken must be treated humanely, 1 sa, 5 sd–21	1: 1.69	2: 1.90	−1.55	0.21	0.001
My chicken welfare knowledge is adequate, 1 sa, 5 sd	1: 2.92	2: 3.27	−1.35	0.26	0.001
Cost of chicken is more important than chicken’s welfare, 1 sa, 5 sd	1: 3.33	2: 2.63	1.73	5.65	0.001
**Consumption**					
Chicken consumption, 1 never, 6 daily	1: 3.61	2: 3.93	−1.06	0.35	0.049
		3: 4.00	−1.87	0.15	0.017
The importance of labelling chicken kept, 1 VI, 5 NI	1: 2.26	4: 3.55	−2.22	0.11	0.001
Need information on chicken welfare, 1 yes, 2 Dk, 3 no	1: 1.49	2: 2.21	−1.91	0.15	0.00
		4: 1.88	−1.64	0.19	0.013
Buy chicken with accredited labelling, 1 yes, 2 no	1: 1.36	4: 1.66	1.67	5.28	0.025

Vg = very good, vb = very bad, vu = very unacceptable, va = very acceptable, sa = strongly agree, sd = strongly disagree, T = true (1), 2 = unsure/do not know (DK), F = False (3); VI = very important; NI = not important (5).

**Table 8:** Significant differences in attitudes of respondents towards meat chicken welfare and consumption of respondents according to marital status. Means are shown for single, no children (referent group, 1) and the comparative groups, single with children (group 2), married/de facto, no children (group 3), married/de facto with children (group 4) and widowed (group 5), as well as coefficients of the regression, odds ratios and *p* values.

Questions and Response Options	Single, no Children (Referent)	Comparative Group	Coefficient	Odds Ratio	*p* Value
**Knowledge**					
Understanding chicken production system (1 little K to 4 expert)	1: 0.98	3: 1.60	−1.39	0.25	0.001
**Attitude**					
Chicken welfare not protected by government standards, 1 sa–5 sda	1: 2.89	3: 2.23	1.17	3.22	0.009
Chicken welfare on farm, 1 vg–5 vb	1: 2.95	5: 2.56	4.22	68.26	0.001
Chicken Welfare during transport 1 vg–5 vb	1: 4.36	2: 4.98	−1.32	0.27	0.009
		4: 3.55	0.71	2.03	0.023
		5: 3.73	2.12	8.33	0.009
Abattoir welfare rating, 1 vg–5 vb	1: 4.58	3: 4.21	−1.17	0.31	0.01
		5: 2.82	4.26	70.89	0.001
Unstunned birds at abattoir, 1 vun–5 va	1: 2.31	5:3.37	−2.17	0.11	0.008
Abattoirs slaughter birds without stunning, 1 vun–5 va	1: 2.41	3: 2.88	−1.14	0.32	0.008
		5: 1.50	2.02	7.55	0.03
Chickens must treated humanely, 1 sa–5 sd	1: 1.48	2: 2.15	−2.05	0.13	0.001
		4: 1.72	−1.12	0.33	0.003
**Consumption**					
My chicken welfare knowledge sufficient 1 sa–5 sd	1: 3.01	5: 2.00	2.19	8.90	0.009
Chicken consumption rate, 1 never, 6 daily	1: 3.70	5: 3.27	3.65	38.55	0.000
The importance of chicken rearing system on 1 vi–5 ni	1: 2.06	4: 2.47	0.75	0.47	0.02
Information on chicken welfare 1 yes, 3 no	1: 1.27	2: 2.00	−3.49	0.03	0.001
		3: 1.48	−1.53	0.22	0.009
		4: 1.63	−1.64	0.19	0.001
		5: 2.00	−3.72	0.02	0.001
Willing to pay how much more for animal welfare rating, 1 no money–5 whatever it takes	1: 2.33	2: 3.67	−2.76	0.06	0.02
		3: 3.32	−1.69	0.18	0.07
		4: 2.35	0.15	1.16	0.56
		5: 5.00	−22.3	0.00	0.00

Vg = very good, vb = very bad, vun = very unacceptable, va = very acceptable, sa = strongly agree, sd = strongly disagree, T = true (1), Unsure/Do Not Know (2), F = False (3); K = knowledge, vi = very important; ni = not important (5).

**Table 9:** Differences between religion groups (Christian, Group 1), compared with other groups, Jewish (Group 2), Hindu (Group 3), Buddhist (Group 4), Muslim (Group 5), Atheist (Group 6) and others (Group 7).

Questions and Response Options	Christian Group	Comparative Groups	Coefficient	Odds Ratio	*p* Value
**Attitude**					
Chicken welfare on farm, 1 vg–5 vb	1:5.22	5:3.54	5:−1.34	0.26	0.003
		6:3.71	6:−0.83	6:0.43	6:0.019
Unstunned birds at abattoir, 1 vu–5 pa	1:2.88	2:2.58	2.08	7.96	0.011
		5:3.08	−1.009	0.36	0.020
		6:2.78	0.95	2.59	0.005
Chicken welfare not protected by government standards, 1 sa–5 sd	1:2.89	6:3.00	1.03	2.79	0.021
Chicken welfare during transport, 1 vg–5 vb, 6 us	1:3.63	6:4.11	−3.71	0.49	0.03
Abattoir welfare rating, 1 vg–5 vb, 6 us	1:3.91	5:4.14	−1.28	0.28	0.006
		6:4.04	−1.00	0.37	0.007
Abattoirs slaughter birds without stunning, 1 vu–5 pa	1:2.88	5:3.78	−1.80	0.16	0.001
Chicken must be treated humanely, 1 sa–5 sd	1:1.43	2:1.04	2.39	10.94	0.036
		5:0.89	2.41	11.12	0.002
		6:0.98	1.79	5.97	0.001
My chicken welfare knowledge is sufficient for food choice, 1 sa–5 sd, 6 us	1:2.45	4:2.45	−2.27	0.10	0.045
		5:2.09	1.52	4.62	0.001
		6:2.18	1.42	4.16	0.001
Cost of chicken is more important than chicken’s welfare, 1 sa–5 sd, 6 us	1:4.14	2:4.92	−2.93	0.05	0.000
		5:4.77	1.12	0.33	0.013
		6:4.58	−0.79	0.45	0.022
**Consumption/labelling**					
The importance of labelling chicken kept, 1 vi–5 ni, 6 us	1:1.97	5:1.29	2.60	13.49	0.001
		6:1.66	1.10	3.00	0.002

Ni, not important, vg = very good, vb = very bad, vi = very important, vu = very unacceptable, va = very acceptable, sa = strongly agree, sd = strongly disagree, us = unsure

**Table 10:** Significant effects of age on responses.

	Coefficient	Odds Ratio	*p* Value
Willing to pay more for animal welfare rating, 1 yes, 2 no	−0.39	0.68	0.0001
Which kind of chicken products are you most likely to buy: products with heart foundation tick	−0.38	0.69	0.03
chicken portions	−0.35	0.71	0.003
Corn- or whole grain-fed	−0.36	0.70	0.0001

## Appendix A.

**Table A1:** Number and % of respondents responding to eight knowledge questions (*correct answer in italics*).

Questions and Response Options	Number of Respondents	% of Respondents
What type of housing is most commonly used to rear meat chickens in Australia?		
Multi-tier battery cages in barns	207	49.3
No housing, free range on pasture is normal	30	7.1
Single tier battery cages on the floor of barns	81	19.3
*Loose in barns*	*102*	*24.3*
How much space is it usual to give each bird in barns?		
About 1 m^2^	168	38.9
*About the size of a piece of A4 paper [*34*]*	*224*	*51.9*
About 5 m^2^	25	5.8
About 2 m^2^	15	3.5
Housing for egg production chickens is the same as for meat production chickens		
True	84	16.7
*False*	*179*	*35.5*
Don’t know	241	47.8
The sex of a chicken is usually determined from the feathers on their wings		
*True*	*61*	*12.4*
False	133	27.00
Don’t know	299	60.6
Chickens are usually fed food of vegetable origin		
*True*	*197*	*39.2*
False	108	21.5
Don’t know	197	39.2
The usual feed for meat chickens in barns is:		
Hay	24	5.4
*Pelleted cereal feed*	*266*	*59.9*
Cut grass	35	7.9
Household waste food	15	3.4
All of these	104	23.4
What is the normal distance that chickens travel from their place of rearing to the abattoir?		
Up to 5 km	85	20.8
*5 to 100 km*	*187*	*45.7*
100 to 200 km	92	22.5
200 to 500 km	29	7.1
500 km or more	16	3.9
Is it normal practice for meat chickens to be rendered unconscious (stunned) before slaughter?		
*Yes*	*116*	*23.9*
No	279	57.4
Don’t know	91	18.7

## Appendix B.

**Table B1:** Stepwise regression of 21 attitude, consumption and demographic predictors on √K score values for 378 respondents.

	Step 1	Step 2	Step 3	Step 4	Step 5	Step 6	Step 7	Step 8	Step 9	Step 10	Step 11	Step 12	Step 13
Constant	3.35	6.38	5.45	6.64	5.59	5.92	7.48	5.20	6.10	4.95	6.00	5.54	6.52
Self-rated understanding of chicken production systems	1.61	1.74	1.65	1.60	1.52	1.50	1.23	1.20	1.19	1.27	1.24	1.23	1.17
*t*-Value	8.40	9.18	8.96	8.80	8.42	8.61	6.34	6.25	6.26	6.68	6.57	6.55	6.18
*p*-Value	0.000	0.000	0.000	0.000	0.000	0.000	0.000	0.000	0.000	0.000	0.000	0.000	0.000
Highest level of education		−0.65	−0.75	−0.76	−0.80	−0.65	−0.56	−0.48	−0.49	−049	−0.49	−0.45	−0.46
*t*-Value		−4.43	−5.20	−5.40	−5.74	−4.68	−3.99	−3.43	−3.47	−3.57	−3.58	−3.21	−3.30
*p*-Value		0.000	0.000	0.000	0.000	0.000	0.000	0.001	0.001	0.000	0.000	0.001	0.001
Killing chickens that are conscious for religious reasons in Australian abattoirs			0.64	0.95	0.98	0.87	0.88	0.78	0.82	0.81	0.83	0.82	0.81
*t*-Value			5.04	6.43	6.69	6.06	6.22	5.38	5.69	5.69	5.82	5.80	5.77
*p*-Value			0.000	0.000	0.000	0.000	0.000	0.000	0.000	0.000	0.000	0.000	0.000
1% of birds do not get adequately stunned in abattoir practices				−0.70	−0.80	−0.70	−0.77	−0.76	−0.71	−0.81	−0.90	−0.91	−0.81
*t*-Value				−3.91	−4.46	−4.01	−4.42	−4.41	−4.13	−4.72	−5.11	−5.21	−4.42
*p*-Value				0.000	0.000	0.000	0.000	0.000	0.000	0.000	0.000	0.000	0.000
Age					0.44	0.87	0.90	0.92	0.90	0.92	0.90	0.88	0.91
*t*-Value					3.46	5.81	6.07	6.24	6.13	6.33	6.23	6.08	6.26
*p*-Value					0.000	0.000	0.000	0.000	0.000	0.000	0.000	0.000	0.000
Marital status						−0.80	−0.83	−0.83	−0.87	−0.89	−0.94	−0.97	−0.98
*t*-Value						−5.00	−5.22	−5.32	−5.59	−5.77	−6.04	−6.22	−6.32
*p*-Value						0.000	0.000	0.000	0.000	0.000	0.000	0.000	0.000
Self-rated knowledge of chicken welfare is enough to form opinion about buying chicken products							−0.54	−0.62	−0.61	−0.59	−0.54	−0.52	−0.50
*t*-Value							−3.17	−3.62	−3.59	−3.52	−3.21	−3.09	−2.96
*p*-Value							0.002	0.000	0.000	0.000	0.001	0.002	0.003
Number of times per week you eat chicken								0.64	0.60	0.63	0.65	0.63	0.62
*t*-Value								2.88	2.70	2.88	2.98	2.90	2.83
*p*-Value								0.004	0.007	0.004	0.003	0.004	0.005
Type of chicken meat consumers buy									−0.45	−0.56	−0.52	−.55	−0.59
*t*-Value									−2.66	−3.27	−3.05	−3.23	−3.45
*p*-Value									0.008	0.001	0.002	0.001	0.001
Would you purchase a product with accredited labelling?										1.04	1.01	1.00	0.89
*t*-Value										3.13	3.04	3.02	2.64
*p*-Value										0.002	0.003	0.003	0.009
Chicken welfare at the abattoir											−0.22	−0.22	−0.22
*t*-Value											−2.22	−2.13	−2.14
*p*-Value											0.027	0.034	0.033
Place of residence												0.43	0.48
*t*-Value												1.81	1.98
*p*-Value												0.071	0.048
Australian meat chickens not protected by government welfare standards													−0.32
*t*-Value													−1.92
*p*-Value													0.056
S	3.36	3.28	3.18	3.12	3.07	2.98	2.94	2.91	2.89	2.86	2.84	2.83	2.82
R-Sq	15.81	19.99	25.08	28.03	30.27	34.67	36.40	37.80	38.97	40.56	41.34	41.87	42.45

## Appendix C.

Significant differences (*p* < 0.05) in knowledge according to demographics that are not presented in the paper. Mean values are shown for the referent group and the comparative group, as well as Odds Ratio and *p* value for the differences.

**Table C1:** Significant differences between males and females.

	Males	Females	Coefficient	Odds Ratio	*p* Value
Space for each bird in barn, (1, 0.25 m^2^–4, 5 m^2^)	1.61	1.88	−0.71	0.49	0.006
Chickens are usually fed food of vegetable origin (1 T, 2 DK, 3 F)	1.82	2.04	−0.80	0.45	0.001
The usual feed for meat chickens in barns, those answering:					
Cut grass	0.52	0.25	−1.89	0.15	0.001
Hay	0.52	0.10	−2.54	0.08	0.001
Pelleted Cereal food	3.8	2.0	−0.78	0.46	0.02

T = true, DK = Do Not Know, F = False.

**Table C2:** Significant differences according to marital status, between Single, no children (referent group, 1) and the comparative groups, single with children (group 2), married/de facto, no children (group 3), married/de facto with children (group 4) and widowed (group 5).

Single, no Children (Referent)		Comparative Group	Coefficient	Odds Ratio	*p* Value
**Knowledge**					
Space for each barn bird (1, 0.25 m^2^–4, 5 m^2^)	1:1.79	4:1.57	0.72	2.00	0.049
Feather sexing of chicken (1 T, 2 DK, 3 F)	1:2.03	2:2.45	−1.95	0.14	0.001
Chicken food is of vegetable origin (1 T, 2 DK, 3 F)	1:1.86	2:1.15 5:2.1	3.55 −2.69	34.82 0.02	0.001 0.003
Chicken travelling distance to abattoir 1 < 5 km–5500 km +	1:2.62	2:2.16 3:1.94 4:2.20	1.59 1.02	4.87 2.77	0.004 0.03
Stunned meat chicken (*1 yes*, *2 DK*, *3 no)*	1:2.27	5:2.70	1.10 −2.33	3.01 0.10	0.002 0.012

**Table C3:** Significant differences according to religion, between the Referent group: Christian, Group 1, compared with comparative groups, Jewish (Group 2), Hindu (Group 3), Buddhist (Group 4), Muslim (Group 5), Atheist (Group 6) and others (Group 7).

	Christian (Referent)	Comparative Group	Coefficient	Odds Ratio	*p* Value
**Knowledge**					
Understanding chicken production systems	1:1.13	6:1.00	−0.72	0.49	0.037
Space for each barn bird (1, 0.25 m^2^–4, 5 m^2^)	1:1.69	5:1.47	1.17	0.03	0.03
Housing the same for egg & meat production	1:2.18	2:1.47 5:1.82	1.86 1.13	6.44 3.08	0.02 0.02
Chicken travelling distance to abattoir	1:0.72	5:0.54	1.22	0.30	0.01
Meat chickens stunned	1:0.21	5:0.50	1.19	3.3	0.01
